# Why and how do general practitioners teach? An exploration of the motivations and experiences of rural Australian general practitioner supervisors

**DOI:** 10.1186/s12909-015-0474-3

**Published:** 2015-10-29

**Authors:** Gerard Ingham, Jennifer Fry, Peter O’Meara, Vianne Tourle

**Affiliations:** Beyond Medical Education, PO Box 3064, Bendigo, 3554 VIC Australia; College of Science, Health and Engineering, La Trobe Rural Health School, La Trobe University, Bendigo, 3350 VIC Australia; Charles Sturt University, Panorama Avenue, Bathurst, NSW 2795 Australia

**Keywords:** Family practice, General practice, Education, Medical, Graduate, Teaching, Mentor, Motivation

## Abstract

**Background:**

In medical education, a learner-centred approach is recommended. There is also a trend towards workplace-based learning outside of the hospital setting. In Australia, this has resulted in an increased need for General Practitioner (GP) supervisors who are receptive to using adult learning principles in their teaching. Little is known about what motivates Australian GP supervisors and how they currently teach.

**Methods:**

A qualitative study involving semi-structured interviews with 20 rural GP supervisors who work within one Regional Training Provider region in Australia explored their reasons for being a supervisor and how they performed their role. Data was analysed using a thematic analysis approach.

**Results:**

GP supervisors identified both personal and professional benefits in being a supervisor, as well as some benefits for their practice. Supervision fulfilled a perceived broader responsibility to the profession and community, though they felt it had little impact on rural retention of doctors. While financial issues did not provide significant motivation to teach, the increasing financial inequity compared with providing direct patient care might impact negatively on the decision to be or to remain a supervisor in the future.

The principal challenge for supervisors was finding time for teaching. Despite this, there was little evidence of supervisors adopting strategies to reduce teaching load. Teaching methods were reported in the majority to be case-based with styles extending from didactic to coach/facilitator. The two-way collegiate relationship with a registrar was valued, with supervisors taking an interest in the registrars beyond their development as a clinician.

**Conclusion:**

Supervisors report positively on their teaching and mentoring roles. Recruitment strategies that highlight the personal and professional benefits that supervision offers are needed. Practices need assistance to adopt models of supervision and teaching that will help supervisors productively manage the increasing number of learners in their practices. Educational institutions should facilitate the development and maintenance of supportive supervision and a learning culture within teaching practices. Given the variety of teaching approaches, evaluation of in-practice teaching is recommended.

**Electronic supplementary material:**

The online version of this article (doi:10.1186/s12909-015-0474-3) contains supplementary material, which is available to authorized users.

## Background

The application of adult learning theory, with its emphasis on a learner-centred approach, has for some decades in medical education been considered essential to facilitate deeper learning [1]. The teacher must be more than an expert lecturer [2]. Consistent with international trends, general practice training in Australia is transitioning from time or process-based requirements to competency or outcomes-based standards [3–5]. Successful implementation of adult learning and competency-based approaches hinge upon their acceptance and adoption by front-line teachers [6]. Concerns have been raised that even experienced and trained medical teachers may struggle to implement an adult learning approach [7].

Most general practice training in Australia occurs in an ‘apprenticeship model’ [8]. Registrars (trainees) see patients on their own under the supervision of an accredited general practitioner (GP) supervisor whilst working at least 18 months in a training practice [9, 10]. GP supervisors in Australia have a formative role in registrar education with fellowship examinations occurring outside of the practice. Not surprisingly, supportive supervision has been found to be the single most important factor in a registrar’s training experience [11].

There is an increasing number of learners being placed in Australian general practice to meet predicted workforce needs [12]. General practice training places in Australia have more than doubled between 2009 and 2015, placing pressure on the recruitment of GP supervisors [13]. The demand is higher in the area outside of major capital cities where 56 % of general practice training is occurring despite only containing 30 % of the GP workforce [14, 15].

An understanding of why rural Australian GPs choose to become postgraduate general practice training supervisors may help inform recruitment and retention strategies for policy makers and educational administrators at a time when demand is increasing. Similarly, educators charged with faculty development will be interested in whether an adult learning approach is being used in workplace-based learning provided in Australian rural general practice. This study aims to investigate the motivations and educational methods of Australian rural GP supervisors. Why and how do rural Australian GP supervisors teach?

## Methods

A descriptive interpretive qualitative study design using thematic analysis was chosen because of its capacity to increase the depth of understanding of the experiences of GP supervisors [16]. The study design fulfilled the criteria for reporting qualitative research as described by Tong et al. [17]. The research team comprised of a male clinician researcher who is a general practitioner and supervisor (GI), a male academic paramedic researcher with extensive qualitative research experience (POM), a female research assistant with a rural allied health background (JF) and a female researcher with an interest in rural health and education (VT). Only one research member had a pre-existing relationship with participants (GI). This relationship was as a medical educator working within the Regional Training Provider (RTP) responsible for overseeing general practice training in the region. The research was initiated by the lead author in response to concerns about decreasing supervisor capacity in rural Australian general practice training and interest in how supervisors teach within their practices.

### Sampling and recruitment

The research was located in a RTP spread across the states of Victoria and New South Wales which did not include any capital cities within its geographical area as the intent was to report on rural supervisors only. GP supervisors were identified through the database of the RTP. Participants were selected using purposeful sampling to recruit a range of supervisors similar to the known gender and age range of GPs, and GP supervisors in the inner regional and outer regional areas of Australia [14, 18]. Consideration was also given to ensuring the sample included a mix of years of experience supervising, levels of remoteness of geographical locations, practice owners and non-owners, sole supervisors, and supervisors in multi-doctor practices and Aboriginal Medical Services.

Potential participants were invited via email to participate in a 45–60 min, individual, face to face, semi-structured interview which would be audio recorded.

### Interviews

Interview questions were developed by the researchers following a review of the literature [19, 20] and were piloted on two GP supervisors who provided feedback resulting in minor changes to the questions. The two interviewing researchers consulted weekly during data collection to compare findings and progressively reflect on the data and refine the interviewing process.

Each participant provided written consent for the interview to be recorded and transcribed. The semi-structured interview explored each supervisor’s motivations, experience, how they performed their supervision role, along with their insights into how the current model was working (see Additional file 1). One technology failure resulted in an interview not being recorded, but contemporaneous notes made by the researcher were agreed as a true reflection of the discussion by the participant and were included in the data. To maintain anonymity, all participants were subsequently assigned a code prior to the review of the transcripts. Field notes were taken during and immediately after each interview by both researchers.

### Analysis

Three researchers read all transcripts progressively through the data collection stage (JF, GI and VT) with one researcher (POM) reviewing a selection of transcripts. Initial codes were derived inductively from early interviews by one researcher (JF) and a framework approach was used to manage the data [21]. Leximancer Version 4.0 ™, a computer aided qualitative data analysis tool, was used by one researcher (VT) to identify and graphically represent concepts [22]. This enabled comparison with manual coding, with the aim of reducing researcher bias [23]. Both the framework analysis and concepts derived through Leximancer were presented to the research team.

All researchers met three times via telephone conferencing during the data collection phase to critically reflect on the codes and emerging themes and determine when saturation of data was reached. A final face to face meeting to arrive at consensus about the grouping of codes into themes was conducted.

## Results

Twenty supervisors from 28 who were invited agreed to be interviewed, with 18 interviews conducted face to face within their own practice and two over the telephone. No supervisors declined to participate, although eight did not respond to the email invitation. The eight who did not respond did not adversely impact on the attainment of the characteristics being purposefully sought. Interviews were conducted by two of the research team (JF and VT) with 11 interviews in New South Wales and nine in Victoria during July to November 2013.

From an existing database held by the RTP the ages of 18 of the 20 supervisors were established. Two were under the age of 40 years and one over the age of 60 years. From answers to interview questions it was established that four of the 20 supervisors had obtained their primary medical degree outside Australia. Two of the supervisors were in solo practice. Fourteen of the 20 supervisors were male. One supervisor had less than five years of experience as a GP, five had 6–15 years of experience, and 14 supervisors had 16 years or more experience.

Eight themes emerged through analysis of the semi-structured interviews. Four themes related to supervisor motivation and four related to teaching and supervision (see Table 1).

**Table 1:** Themes identified

Supervisor motivation	
1. Personal and professional benefit of being a supervisor	
2. Responsibility to the profession of general practice	
3. Benefits for the GP practice or business	
4. Responsibility to the community	
Teaching and Supervision	
5. Not enough time	
6. Administrative burden	
7. Variety in teaching	
8. Supervisor-registrar relationship	

### Supervisor motivation

#### Theme 1: personal and professional benefit of being a supervisor

GP Supervisors were highly motivated by the personal and professional benefits they received from teaching and mentoring registrars. The majority of supervisors taught because they enjoyed it.

If I didn’t enjoy teaching I would stop … encouraging doctors to stay in the area is kind of a secondary thing. Sup 4

They felt an innate desire to teach. Many identified the influence of family members who were teachers, as well as positive and negative influences from medical teachers in their past.

Supervisors described how the process of teaching helped their own professional development as a GP. Exposure to a learner with recent knowledge and understanding of current practice was valued.

Professionally and personally it’s satisfying. It keeps you in the loop, especially when you are in an isolated, small town you need to keep your skills up. It is good to share the skills. Sup 17

Supervisors identified the reflective nature of postgraduate teaching as enhancing their understanding of general practice.

I learn at least as much from my registrars as they ever learn from me and it is often that I don’t even see teaching as teaching because I think it is often more just talking about things and working out answers to things. So it’s more of a facilitator perhaps than a teacher. Sup 1

Being a teacher gave supervisors a sense of pride. Registrar teaching offered supervisors variety and challenge in the working week. It provided intellectual stimulation.

Financial remuneration was acknowledged as important, but generally did not influence the participants’ decision to supervise. However, several supervisors expressed concern that supervising registrars was not as financially viable as consulting and may eventually become too strong a disincentive to take on or continue supervising.

The amount that I would get from teaching is not the same hourly rate that I would get from consulting and as the gap gets bigger and bigger, as the [supervisor] pay rate stays the same, eventually that is going to start to be an issue. Sup 4

Some supervisors had not investigated the impact of being a supervisor on their business, because they were so committed to the process of teaching.

To be quite honest I have not been through the finances with every registrar in seeing whether we actually make money on registrars or lose money on registrars. My gut feeling is that they are cost neutral. Sup 9

Supervisors, despite not being motivated to perform their role for financial reasons reported they wanted fair compensation, and to feel they were being valued.

#### Theme 2: responsibility to the profession of general practice

Supervisors perceived that being a teacher was a core component of their responsibility to the medical profession, and specifically to the discipline of general practice. Supervisors were compelled by a desire to model and promulgate a high standard of general practice and through this to further the standing of GPs in the community. It was inconceivable to some that they could be a GP without teaching. This sense of responsibility could over-ride doubts some had about their suitability to be a teacher.

Why, why do we all do it? … It was because we took a Hippocratic Oath that said we must pass on knowledge, and we take that very seriously. Sup 11

It is not something that comes naturally and it is not something that I love doing. … [but] I have always had a fairly strong sense of responsibility … to impart knowledge to younger people. Sup 7

#### Theme 3: benefits for the GP practice or business

Whilst, in general, supervisors reported personal benefit or professional responsibilities as the main reason for being a supervisor, providing a workforce was also a motivator.

I’m clear that we don’t make gaining workforce the only reason to get registrars in, but it is an important one. It provides a really useful service and we need to really appreciate that. Sup 13

A number of supervisors reported that having registrars and students in the practice resulted in the development of a learning environment, improving the collegiate relationships and shared learning between all doctors. Registrar placements improved the quality and functioning of the medical clinic. Conversely, some supervisors thought that having registrars could be a negative influence on their practice, particularly if the registrar’s skill was substandard or in the early phases of their placements when the registrar’s efficiency was less.

#### Theme 4: responsibility to the community

Supervisors considered that through their involvement in the GP registrar training program they were improving the standard of healthcare for all Australians.

I think people should be offered the best healthcare …. I think that my part in it would be to help elevate a level of practice in General Practice. Sup 3

There were some supervisors who expressed the opinion that it improved their own practice’s care for the patients in their community.

Registrars tend to spend a little bit more time with people, sometimes uncover things that their usual doctor just might have missed. Sup 1

Rural supervisors were hopeful that registrars would remain in their local area. The issue of retention of GPs in rural areas was a strong theme but supervisors believed they were relatively powerless to influence the registrar’s decision. They perceived that a registrar’s decision to continue to practice in a rural setting post completion of training was more influenced by the registrar’s social situation, their partner’s employment, the existence of educational opportunities for their children, and the presence of cultural supports.

I know that a good proportion of them will only stay in the country if their kids are settled. Sup 5

A sense of frustration was particularly reported by supervisors in towns located close to the capital cities. They felt they were less likely to retain registrars, as often registrars chose to not relocate to the town, but to commute from the nearby city. They considered the resulting lack of connection with the rural community made it less likely that the registrar would return to a rural setting.

None of the registrars have ever talked about wanting to come back here. They all, a few of them because of family, ethnic or religious reasons, very clearly want to get back to the [capital city] suburbs. Sup 9

### Teaching and supervision

#### Theme 5: Not enough time

In response to questions about barriers to supervising, a lack of time was reported to be the most significant impediment to successful supervision. Supervisors struggled to ‘find time’ for both ad hoc (corridor) teaching and planned (tutorial) teaching. This impacted upon their enjoyment of teaching and on their performance as a teacher.

Available time is the main issue. It gets to the teaching on the Wednesday afternoon and I will just have ten minutes to finish my lunch. Sup 12

When you are time pressured you don’t always do the corridor teaching as well as you know that you could, or should, or would like to. You are busy and you are already running late with other things. Sup 1

I think of a lot of the things that I want to teach aren’t taught quickly. Sup 13

For most supervisors, repeated interruptions for supervision and ad hoc teaching were tolerated as part of the role, particularly when registrars were new. They reported that interruptions reduced as the registrar became more proficient and the burden of supervision became less. In contrast, a few supervisors took a more active role in structuring their day to enable them to have time to cope with interruptions. Assisted by their practice managers they modified appointment schedules to reduce their own clinical load, implemented supervisor rosters and involved the broader practice team in supervision to share the load.

Some supervisors provided the required tutorial teaching as a set aside session in practice hours. Others described a more fluid approach to finding the required time and one conducted teaching after hours. Supervisors who reported fewer problems with time had purposely restructured their practice to work in an environment where they had combined adequate time to teach with reduced clinical load.

Being in a job now where I have got time to do a lot of other stuff that is unpressured by appointments is really helpful. Sup 13

#### Theme 6: administrative burden

Supervisors were aware of the burden on their staff associated with the administration of the training program and the orientation of new registrars to the practice. Supervisors within small practices commented on the difficulty of managing the registrar supervision requirements when they took leave. Supervisors also reported their practices were impacted by ‘fallow periods’ when a period with no registrars followed a six month registrar placement.

Turning the tap on and off in a smallish practice can be a challenge. We have got to keep enough patients to be able to keep the registrars fuelled. Sup 2

#### Theme 7: variety in teaching

Most supervisors reported using case-based teaching but the teaching styles described by the supervisors were diverse. Whilst some supervisors reported modifying their teaching according to the learning needs of their registrars, others tended to deliver, even if not written down as such, a predetermined syllabus in their tutorials.

It has swung too much the other way. When we went through it was all didactic. Then it swung to this self-directed learning, and do-it-yourself workshops and things, and they have got big holes in their knowledge. So now we are coming back to a bit of didactic [teaching] and then applying that knowledge in the case studies. Sup 11

For other supervisors the teaching and learning was consciously woven into the fabric of everyday practice. Registrar learning arose dynamically from clinical and practical experience in the workplace.

The impartation of knowledge, skill and wisdom goes on all of the time and whether it be over lunch, whether it be corridor discussion, whether it be call me in and talk over a situation, whether it be phone me, or whether we sit down and do some tutorials. Sup 13

Teaching basic medical knowledge and skills was considered by many supervisors to not be their role. They believed there were superior learning resources available for this. Registrars were successful graduates seeking practical experience and the strengths that supervisors brought to the training program were in facilitating learning in a real world setting and in developing registrars’ consultation skills, clinical reasoning and professional behaviour.

I have developed my teaching away from topic teaching to much more case-based, including video review, random case analysis, and audit of referrals or investigations. The registrars do a lot of their own topic learning and I really focus more on consultation techniques and using their knowledge in the context of general practice. Sup 1

Supervisors described the use of reflective questioning to explore clinical reasoning and the provision of balanced and learner-centred feedback. They had difficulty articulating how they were teaching. Some doubted they were teaching in the correct manner.

I can’t remember whether there is a formal term for reflect the question back on the person who has asked it. You know, what do you think so far and how are you going to work out whether you are right or wrong? What is your next step? and so on. Sup 12

Some teaching methods tended to polarise supervisors. Teaching by review of video-recorded consultations was either embraced positively, or wholeheartedly rejected. Video equipment difficulties were the major reason for rejecting the method but some supervisors also had doubts regarding the pedagogical value of reviewing video-recorded consultations compared with direct observation of consultations. Whilst supervisors thought video observation of consultations was more valuable in promoting registrar self-reflection, direct observation of consultations was preferred as it provided stronger assurance that their registrars’ patients were being safely managed as the registrar did not have the ability to select the consultations for review.

I still do a lot of sitting in because I think that you have just got to do that for safety sake. Sup 10

Review of medical records was used by several supervisors. This occurred opportunistically when a patient attended to see them after previously seeing a registrar. A random review of recent registrar medical records was conducted by some supervisors during tutorials and used as a springboard for broader discussion.

Most supervisors reported using other members of their practice team in teaching. The practice team were seen to have a role in supporting the registrar. They provided orientation to the practice, community and the healthcare system, and also helped to identify the registrar in difficulty. Clinical teaching was also provided by practice nurses and allied health practitioners within the practice when available.

#### Theme 8: supervisor-registrar relationship

Supervisors described the significant relationship between themselves and the registrar as a two-way benefit and spoke of the collegiality they developed with their registrar. The supervisors had a deep understanding and interest in their registrars’ progression, extending beyond improvement as a clinician.

I get a lot of satisfaction out of seeing young ones grow in their confidence as well as their expertise. I’m very, very strong in looking after them and making sure how they are doing rather than just how they are going professionally. Sup 13

In practices that had moved to having more supervisors involved in registrar teaching, supervisors noted the less frequent contact between themselves and their registrar. Whilst some supervisors regretted the reduced depth of relationship, the exposure to multiple supervisors was considered to have benefits for the registrar.

It is not a continuing one to one relationship and so I have pondered about whether that is better or not. The registrars who have had three teachers have reflected on it saying they like having three teachers. Sup 2

I think that it is healthy for the registrar to go and work under other doctors as well so that they might have a different view of things and the registrar can learn from that. Sup 3

## Discussion

We found that rural GP supervisors are strongly motivated by the personal and professional benefits of being a teacher as well as the benefits to the training practice. Similar motivations have been reported in teachers of both medical undergraduates and postgraduates [24–28]. Our findings support enhancing supervisor recruitment by giving recognition to teaching as a professional development activity that provides personal enjoyment for supervisors while meeting their sense of professional responsibility [27].

Studies have found that teaching medical students can have either positive [28] or negative [29] effects on training practice staff morale. We found that teaching GP registrars has a generally positive impact. This difference may reflect the stimulus provided by the presence of more advanced learners in the practice as well as the contribution of registrars to practice workforce. Educational institutions should consider including the positive impact of registrars on staff morale in marketing strategies.

As with teachers in other health disciplines [30], financial rewards were not a significant motivator for the GP supervisor role. A financial analysis of registrar training in Australia has found small, if any, financial benefit to the practice [31]. Our participants provide a note of caution that deteriorating remuneration compared to clinical work may eventually impact upon recruitment and retention of supervisors. A questionnaire based survey of practices in another Australian RTP reported that increasing teaching subsidies would be an important contributor to increasing teaching capacity [26].

A significant reason for taking on the supervisor role was the hope that some registrars would remain permanently in the practice and solve succession problems for the practice. Despite this reason for supervising, supervisors felt they had little influence upon the registrar’s ultimate decision to remain in rural areas. Other contextual factors have been reported to have greater influence [32–34]. In Australia there is expected to be an increasing mismatch between available training places and doctors seeking training due to increasing numbers of Australian and international medical graduates [35]. The availability of registrar training places might be further threatened if other (non-registrar) doctors were more willing to remain permanently in training practices.

The difficulty in finding time for teaching was a strong theme that has also been consistently found in other papers [27, 29, 36, 37]. In general, our study participants had not made significant changes to their clinical work to accommodate teaching. A few supervisors had modified their appointment schedules, developed supervisor rosters and involved the broader practice team in supervision, but there was little reporting of the adoption of other strategies that have been proposed to reduce supervisor teaching workload such as shared learning sessions [38] or the use of registrars as teachers [39]. Planning is important for effective learning and supervision [19, 40], but changing workplace culture is often an impediment to introducing new models of learning [41]. More specific assistance to training practices to help them think laterally for solutions [37] and implement reform would appear necessary.

Supervisors described a variety of teaching styles. Despite often being unable to explain how they were teaching in the language of education theory, they described responding to registrar learning needs as they arose in the workplace and using questioning techniques to explore understanding and promote further learning. This behaviour is consistent with a learner-centred adult education approach. In other studies, GP teachers have been found to have low regard for and use of adult learning principles [42, 43]. This was less evident in our participants and perhaps reflects the Australian postgraduate training environment and the compulsory attendance of GP supervisors at supervisor training. Of course, what GP supervisors report doing and what they actually do may well be different. There is a lack of empirical evidence about the quality and effectiveness of in-practice, workplace-based teaching for general practice [19, 20].

Supervisors valued the relationship with their registrars and took an interest in them that extended beyond their clinical advancement. The importance of the ethos of caring for the learner in general practice training and providing the right mix of challenge and support has been reported previously [20, 42, 44]. As practices become larger and more learners are present, the relationship and support of the registrar will depend less on individual supervisors and more upon the culture within the practice as a whole. The involvement of practice managers and other members of the practice supervision team in professional development activities would appear prudent in this context.

This study explored the motivations and teaching practices of rural GP supervisors of GP registrars. The use of semi-structured interviews, the maintenance of anonymity and the involvement of researchers from outside the general practice vocational training field (JF, VT, POM) has contributed to the veracity of the supervisors’ responses and their interpretation. As the study reports on GP supervisors working within one Regional Training Provider which has a rural footprint, the findings may not be transferrable, particularly to urban supervisors. We have not investigated the reasons why GPs choose not to become supervisors. In respect to supervisor teaching styles and activities, there was no observation of practice to confirm what supervisors report doing.

## Conclusions

This study confirms that GP Supervisors are motivated by the personal and professional benefits of teaching, their altruistic responsibility to the profession of general practice, and the improvements that teaching brings to their practice and their community. Financial rewards are not an important motivator. Recruitment strategies for new supervisors should highlight the personal and professional benefits gained through supervising.

The supervisors interviewed reported struggling with the competing demands of clinical practice and teaching. Supervisor training should address the knowledge and skills required to implement time-efficient teaching techniques. General Practice training organisations should involve themselves with the entire practice and consider providing management expertise and support to individual practices to overcome impediments to change and to help them develop supportive supervision and a learning culture.

Our study suggests many, but not all, supervisors are adopting an adult learning approach. There is a need for further empirical research and evaluation of in-practice teaching and supervision so that it might guide further supervisor professional development and support the wider use of an adult learning approach in general practice training posts.

### Ethics approval

Ethics approval was granted by LaTrobe University Faculty Human Ethics Committee.

## Additional file

Additional file 1:
**Semi structured interview format.** (DOCX 18 kb)

## Abbreviations

GP: General practitioner
RTP: Regional training provider

## References

[1] Spencer JA, Jordan RK (1999). Learner centred approaches in medical education. BMJ.

[2] Crosby RM, Harden J (2000). AMEE guide No 20: the good teacher is more than a lecturer - the twelve roles of the teacher. Med Teach.

[3] Roberts RG, Hunt VR, Kulie TI, Schmidt W, Schirmer JM, Villanueva T (2011). Family medicine training—the international experience. Med J Aust.

[4] Iobst WF, Sherbino J, Cate OT, Richardson DL, Dath D, Swing SR (2010). Competency-based medical education in postgraduate medical education. Med Teach.

[5] RACGP Vocational Training Standards. [http://www.racgp.org.au/education/rtp/vocational-training-standards]. Accessed 1 July 2015.

[6] Dath D, Iobst W (2010). The importance of faculty development in the transition to competency-based medical education. Med Teach.

[7] Kogan JR, Conforti LN, Bernabeo EC, Durning SJ, Hauer KE, Holmboe ES (2012). Faculty staff perceptions of feedback to residents after direct observation of clinical skills. Med Educ.

[8] Trumble SC (2011). The evolution of general practice training in Australia. Med J Aust.

[9] Registrar Handbook. [http://www.racgp.org.au/download/Documents/Fellowship/registrar-handbook.pdf]. Accessed 1 September 2015.

[10] Vocational Training. [https://www.acrrm.org.au/vocational-training]. Accessed 1 September 2015.

[11] Findlay D (2014). Quality training practices - the registrars’ perspective.

[12] HW2025 Volume 1. [https://www.hwa.gov.au/our-work/health-workforce-planning/health-workforce-2025-doctors-nurses-and-midwives]. Accessed 1 July 2015.

[13] Australian General Practice Training Program. [https://www.health.gov.au/internet/main/publishing.nsf/Content/work-st-agpt]. Accessed 21 June 2015.

[14] Medical workforce 2011. National health workforce series. [http://www.aihw.gov.au/publication-detail/?id=60129542627]. Accessed 1 July 2015.

[15] GPET Annual Report 2013. [http://www.gpet.com.au/About-Us/Annual-Report]. Accessed 1 July 2015.

[16] Vaismoradi M, Turunen H, Bondas T (2013). Content analysis and thematic analysis: implications for conducting a qualitative descriptive study. Nurs Health Sci.

[17] Tong A, Sainsbury P, Craig J (2007). Consolidated criteria for reporting qualitative research (COREQ): a 32-item checklist for interviews and focus groups. Int J Qual Health Care.

[18] Kinsella P, Wood J (2008). GP supervisors-their professional development and involvement in assessment. Aust Fam Physician.

[19] Kilminster S, Cottrell D, Grant J, Jolly B (2007). AMEE guide No. 27: effective educational and clinical supervision. Med Teach.

[20] Wearne S, Dornan T, Teunissen PW, Skinner T (2012). General practitioners as supervisors in postgraduate clinical education: an integrative review. Med Educ.

[21] Gale NK, Heath G, Cameron E, Rashid S, Redwood S (2013). Using the framework method for the analysis of qualitative data in multi-disciplinary health research. BMC Med Res Methodol.

[22] Smith AE, Humphreys MS (2006). Evaluation of unsupervised semantic mapping of natural language with Leximancer concept mapping. Behav Res Methods.

[23] Penn-Edwards S (2010). Computer aided phenomenography: the role of leximancer computer software in phenomenographic investigation. Qual Rep.

[24] Dahlstrom J, Dorai-Raj A, McGill D, Owen C, Tymms K, Watson DA (2005). What motivates senior clinicians to teach medical students?. BMC Med Educ.

[25] May M, Mand P, Biertz F, Hummers-Pradier E, Kruschinski C (2012). A survey to assess family physicians’ motivation to teach undergraduates in their practices. PLoS One.

[26] Laurence CO, Black LE (2009). Teaching capacity in general practice: results from a survey of practices and supervisors in South Australia. Med J Aust.

[27] Thomson J, Haesler E, Anderson K, Barnard A (2014). What motivates general practitioners to teach. Clin Teach.

[28] Hartley S, Macfarlane F, Gantley M, Murray E (1999). Influence on general practitioners of teaching undergraduates: qualitative study of London general practitioner teachers. BMJ.

[29] Sturman N, Régo P, Dick M-L (2011). Rewards, costs and challenges: the general practitioner’s experience of teaching medical students. Med Educ.

[30] Bonetti D, Ross J, Stewart S, Clarkson J (2007). What influences intention to become a postgraduate trainer?. Br Dent J.

[31] Laurence CO, Black LE, Karnon J, Briggs NE (2010). To teach or not to teach? A cost-benefit analysis of teaching in private general practice. Med J Aust.

[32] Chan BT, Degani N, Crichton T, Pong RW, Rourke JT, Goertzen J (2005). Factors influencing family physicians to enter rural practice: does rural or urban background make a difference?. Can Fam Physician.

[33] Henry JA, Edwards BJ, Crotty B (2009). Why do medical graduates choose rural careers?. Rural Remote Health.

[34] Campbell DG, Greacen JH, Giddings PH, Skinner LP (2011). Regionalisation of general practice training -are we meeting the needs of rural Australia?. Med J Aust.

[35] Australia’s Future Health Workforce - Doctor’s report. [http://www.health.gov.au/internet/main/publishing.nsf/Content/australias-future-health-workforce-doctors]. Accessed 21 June 2015.

[36] Wearne SM (2003). Pilot study on the factors that influence learning by general practice registrars in central Australia. Rural Remote Health.

[37] Pearce R, Laurence CO, Black LE, Stocks N (2007). The challenges of teaching in a general practice setting. Med J Aust.

[38] Ahern CM, van de Mortel TF, Silberberg PL, Barling JA, Pit SW (2013). Vertically integrated shared learning models in general practice: a qualitative study. BMC Fam Pract.

[39] Anderson K, Thomson J (2009). Vertical integration - reducing the load on GP teachers. Aust Fam Physician.

[40] Ingham G (2012). Avoiding ‘consultation interruptus’ - a model for the daily supervision and teaching of general practice registrars. Aust Fam Physician.

[41] Matthews LR, Pockett RB, Nisbet G, Thistlethwaite JE, Dunston R, Lee A (2011). Building capacity in Australian interprofessional health education: perspectives from key health and higher education stakeholders. Aust Health Rev.

[42] Cantillon P, de Grave W (2012). Conceptualising GP teachers’ knowledge: a pedagogical content knowledge perspective. Educ Prim Care.

[43] Longman C, Temple-Smith M (2013). General practice registrar observation of their supervisors in consultation - what is the educational value?. Aust Fam Physician.

[44] Bayley SA, Magin PJ, Sweatman JM, Regan CM (2011). Effects of compulsory rural vocational training for Australian general practitioners: a qualitative study. Aust Health Rev.

