# Supplementary material for: Why and how do general practitioners teach? An exploration of the motivations and experiences of rural Australian general practitioner supervisors
Source: BMC Med Educ. 2015 Oct 29;15:190. doi: 10.1186/s12909-015-0474-3 (PMC4625577; doi:10.1186/s12909-015-0474-3)
Supplement: Additional file 1: — Semi structured interview format. (DOCX 18 kb) [file 12909_2015_474_MOESM1_ESM.docx]

Semi structured interview format:

1. Self and practice demographics
2. History as a GP supervisor
3. Can you describe a typical progression of a registrar through your practice?
4. Can you identify both the benefits and disadvantages of being a GP supervisor?

- For the practice
- For you personally or professionally

1. Could you describe what motivated you to become a supervisor in the first place, and now why you stay being a supervisor?
2. Does being a supervisor have a positive or negative impact on your own clinical practice?
3. Can you describe the activities you tend to use as teaching tools?

- Follow up questions if needed: Do you provide face to face feedback to registrars or rely on the RTP feedback paperwork?
- Do you tend to schedule teaching, or is it opportunistic?

1. For you, which tools work best?
2. How do you rate yourself as a teacher/supervisor?

- What barriers do you have for best practice as a supervisor

1. What criteria do you use to assess yourself?
2. How do you respond to the proposal for an alternative teaching model:

“*An alternative supervision model is being proposed in which a supervisor would still be responsible for supervision of the registrar's clinical practice but teaching sessions would be provided by educators from the Regional Training Provider*”

1. What advantages and disadvantages do you see to this model?
2. Can you see that it might work for some practices more than others? What sorts of practices are these?
3. What do you think works well in the GP Registrar Program, and what is not working well?
4. Is there anything further you would like to add to the conversation about GP teaching and your role as a supervisor?
